# Influence of Olive Oil Components on Ion Channels

**DOI:** 10.3390/molecules30163336

**Published:** 2025-08-11

**Authors:** Hascibe Mijares-Andrade, Ismael Carreño-Diaz, Osmel La-Llave-Leon, Ivan Meneses-Morales, Estela Ruiz-Baca, Angelica Lopez-Rodriguez

**Affiliations:** 1 Instituto de Investigación Científica, Universidad Juárez del Estado de Durango, Durango 34000, Mexico; hasmija@hotmail.com (H.M.-A.); olallavel@ujed.mx (O.L.-L.-L.); 2 Facultad de Ciencias Químicas, Universidad Juárez del Estado de Durango, Durango 34120, Mexico; ismaelacd@hotmail.com (I.C.-D.); ivan.meneses@ujed.mx (I.M.-M.); eruiz@ujed.mx (E.R.-B.)

**Keywords:** oleic acid, protein traffic, membrane, lipids, polyphenols, metabolism, olive oil

## Abstract

Olive oil, a cornerstone of the Mediterranean diet, contains a saponifiable lipid fraction rich in oleic acid, and a non-saponifiable fraction composed of minor bioactive constituents such as squalene, vitamin E, oleuropein aglycone, hydroxytyrosol, oleocanthal, and oleacein, among other phenolic and triterpenic compounds. These components are well-documented for their cardiovascular, anti-inflammatory, antioxidant, and neuroprotective activities. This review explores the physiological relevance of olive oil lipids and their derivatives on cellular membranes and ion transport systems, by combining biochemical and electrophysiological insights. We discuss how oleic acid and its metabolites influence membrane lipid composition, modulate fluidity, and reorganize lipid rafts—key elements for the proper localization and function of ion channels. Additionally, we examine evidence showing that several olive oil components regulate ion channels such as TRP, potassium, calcium, and chloride channels, as well as other transporters, thereby influencing ionic homeostasis, oxidative balance, and signal transduction in excitable and non-excitable cells. By combining these findings, we propose a conceptual framework in which olive oil lipids and their derivatives act as multimodal regulators of bioelectrical signaling. By modulating cell membrane dynamics, these functional molecules help maintain cellular communication and homeostasis. This integrative view not only strengthens our understanding of olive oil’s health-promoting effects but also opens new avenues for targeting ion-regulatory mechanisms in metabolic, cardiovascular, and neurological diseases.

## 1. Olive Oil, Its Components, and Their Role in Human Health

Olive oil, derived from the fruit of the Olea europaea tree, is far more than a flavorful culinary fat. For over 4000 years—since its earliest documented uses in ancient Egypt, Greece, and Rome—olive oil has been valued for culinary, medicinal, cosmetic, and spiritual purposes [1]. Recent research has elucidated how its constituents interact with cellular membranes and modulate ion channels, suggesting potential therapeutic applications in cardiovascular, neurological, and inflammatory disorders [2]. Its unique composition, rich in monounsaturated fats and bioactive phenolics, underpins both its sensory appeal and health-promoting properties.

### 1.1. Constituents of Olive Oil

Olive oil is chemically composed of two principal fractions: the major saponifiable fraction, which accounts for approximately 98–99% of the oil and consists primarily of triacylglycerols rich in monounsaturated fatty acids, and the minor non-saponifiable fraction, which includes a diverse array of bioactive compounds such as phenolics, tocopherols, sterols, squalene and triterpenes. Additionally, certain functional constituents like pigments and volatile compounds, although not strictly classified under these two chemical fractions, contribute significantly to the oil’s nutritional value and sensory characteristics. Table 1 summarizes the main chemical classes and representative compounds identified in each fraction [3,4,5,6,7,8].

### 1.2. Absorption, Metabolism and Excretion of Olive Oil Components

**Figure 1:**
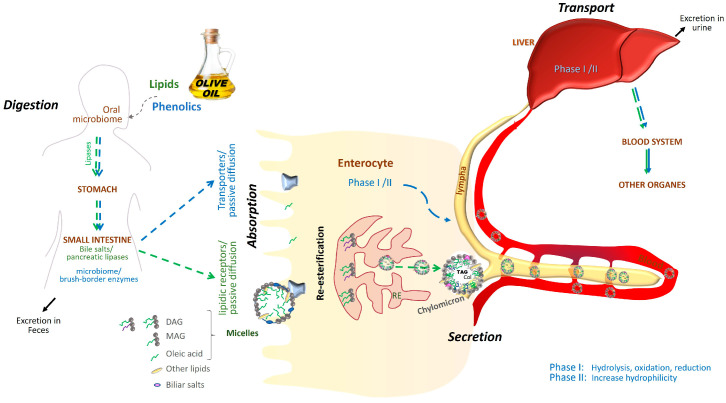
Biochemical pathway of olive oil absorption. Overview of the absorption and metabolism of lipidic and phenolic compounds from olive oil. After ingestion, olive oil components follow distinct yet parallel metabolic routes. Lipidic compounds—primarily oleic acid in triacylglycerol form—undergo enzymatic digestion, absorption in the small intestine, re-esterification, and incorporation into chylomicrons for systemic distribution. Phenolic compounds, such as oleuropein aglycone, hydroxytyrosol, and oleocanthal, are initially transformed by oral and gut microbiota, then absorbed and subjected to phase I and II metabolic reactions that enhance their bioavailability and facilitate tissue distribution. Green arrows depict the pathway of lipid compounds; blue arrows show the phenolic compound route.

Figure 1 summarizes the metabolic fate of lipidic and phenolic compounds derived from olive oil after ingestion, illustrating their parallel absorption and metabolic routes, highlighted by green and blue arrows, respectively. These visual pathways reflect the sequential processes involved in the digestion of olive oil components. The digestion of the primary lipid components, predominantly triacylglycerols, is begun by oral lipases and is subsequently followed by (1) emulsification via the action of bile salts and (2) hydrolysis by gastric and pancreatic lipases into free fatty acids and monoacylglycerols. These products form micelles, facilitating their absorption in the small intestine [9]. Within enterocytes, they are re-esterified into triacylglycerols and packaged into chylomicrons, which are secreted into the lymphatic system, enter the bloodstream, and reach the liver. From there, they circulate to peripheral tissues, where triacylglycerols are hydrolyzed and oleic acid is taken up by cells, as represented by the green pathway in Figure 1. Once in circulation, oleic acid can be taken up by peripheral tissues, where it may be oxidized for energy, stored in adipose tissue, or incorporated into membrane phospholipids, modulating fluidity and signal transduction [9,10,11].

Interestingly, oleic acid’s incorporation into endoplasmic reticulum phospholipids plays a role in vesicle formation and protein trafficking. Oleic acid can be incorporated into endoplasmic reticulum (ER) phospholipids, influencing membrane curvature and promoting vesicle budding, as supported by studies on lipid trafficking and membrane dynamics [12,13]. Membrane vesicles enriched in oleic acid have been shown to participate in the transport of newly synthesized proteins from the ER to the Golgi apparatus for post-translational modification, and may subsequently fuse with other cellular compartments, including the plasma membrane and mitochondria. Although these processes have not been directly studied in the context of olive oil consumption, they provide a mechanistic basis for understanding how dietary oleic acid may influence intracellular protein trafficking [14]. The monounsaturated structure of oleic acid enhances membrane fluidity by disrupting tight phospholipid packing, which facilitates protein transport and vesicle fusion [15,16,17,18].

Beyond fatty acid metabolism, olive oil contains phenolic compounds that undergo a complex and tightly regulated absorption and biotransformation process in the human body. Glycosylated phenolic compounds are initially modified by the oral microbiota, as indicated in the initial step on the blue pathway in Figure 1, and undergo limited hydrolysis in the stomach. In the small intestine, brush-border enzymes such as β glucosidase and lactase phlorizin hydrolase release aglycones, which can be absorbed via passive diffusion or transporter-mediated mechanisms. After absorption, these phenolics undergo phase I and II metabolism—including methylation, sulfation, and glucuronidation, resulting in diverse bioactive metabolites with a specific tissue distribution [19,20,21,22,23,24].

Of particular interest, oleocanthal metabolism and pharmacokinetics have been recently characterized in detail in vivo, demonstrating its absorption, systemic circulation, and formation of distinct metabolites in humans [25]. Among the phenolic compounds derived from olive oil, oleuropein aglycone (OLEA) and oleocanthal are of particular relevance due to their chemical structure, oil-specific formation, and physiological effects. OLEA is generated during the malaxation step of oil extraction by β-glucosidase activity, which hydrolyzes oleuropein originally present in the olive fruit and leaves. Once ingested, OLEA is absorbed mainly in the small intestine, likely through passive diffusion facilitated by its lipophilic nature, and subsequently undergoes phase II metabolism [26,27,28]. In parallel, oleocanthal, a dialdehydic secoiridoid unique to extra virgin olive oil, is also formed during malaxation from the enzymatic conversion of ligstroside aglycone [29,30]. It is absorbed in the small intestine and biotransformed via glucuronidation, sulfation, and methylation in enterocytes and hepatocytes, producing circulating metabolites detectable in human plasma and urine shortly after ingestion [20,30]. These findings support the systemic bioavailability and potential functional roles of both compounds following dietary intake of extra virgin olive oil.

Hydroxytyrosol and tyrosol, major phenolic compounds in olive oil, are efficiently absorbed through passive diffusion and, to some extent, active transport [28]. In contrast, oleuropein, which is more abundant in olive leaves and unripe olives, is poorly absorbed in its intact form. It undergoes hydrolysis by intestinal enzymes and microbiota to yield hydroxytyrosol and its aglycone, enhancing its bioavailability [31,32,33,34,35].

Absorption efficiency varies with molecular characteristics. Low-molecular-weight compounds, such as gallic acid (present in trace amounts in olive oil) are easily absorbed, whereas high-molecular-weight polyphenols, such as proanthocyanidins (typical of berries and cocoa, but not detected in olive oil), require prior degradation. Lipophilic aglycones diffuse freely across enterocytes, while glycosylated and hydrophilic phenolics, such as quercetin glycosides and anthocyanins from other sources, rely on transporters like SGLT1 and GLUT1/3 [19,20].

Unabsorbed phenolics that reach the colon are metabolized by gut microbiota through decarboxylation, hydrolysis and dehydroxylation, producing smaller bioactive metabolites. For example, flavonols are converted into hydroxyphenylacetic acids, flavanols into phenylvalerolactones, and anthocyanins into benzoic acid derivatives. These microbial metabolites may undergo phase I and II reactions in enterocytes and subsequently in the liver, including oxidation, methylation, glucuronidation, and sulfation, which significantly increase their water solubility and facilitate plasma transport and renal excretion [19,20,36,37,38]. Peak plasma levels of hydroxytyrosol and its related metabolites are typically reached within 30 to 60 min post-ingestion, with 5–10% of the ingested dose excreted in urine within 24 h [34,39].

Interestingly, molecular studies have shown that OLEA interacts preferentially with negatively charged phospholipids such as POPG over zwitterionic ones like POPC, potentially affecting its localization in cellular membranes and its influence on membrane fluidity and protein distribution [40]. Moreover, experimental evidence demonstrates that olive oil phenolic compounds, including OLEA and hydroxytyrosol, can be incorporated into lipid-based biological structures, such as low-density lipoproteins (LDLs), and protect them against oxidative damage [41], further supporting their membrane-targeting capacity.

## 2. Influence of Olive Oil Derivatives on Ion Channels

**Figure 2:**
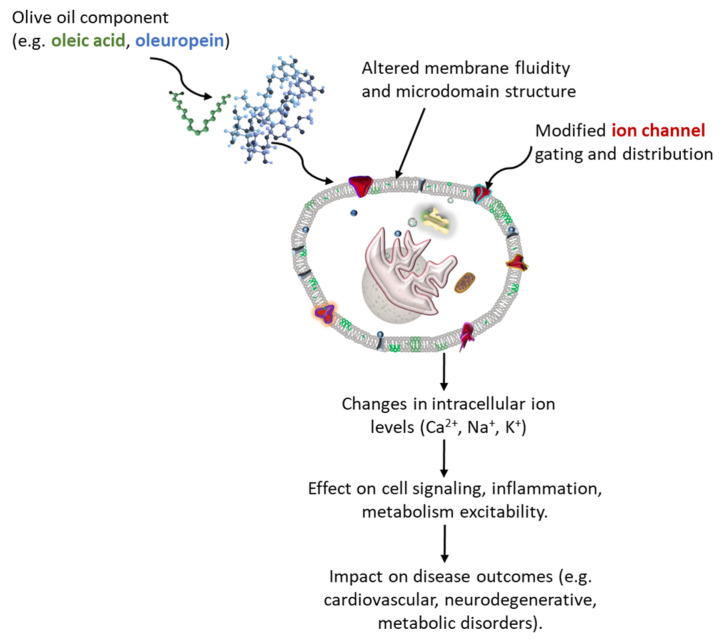
Proposed mechanism by which olive oil components modulate ion flux and contribute to health outcomes. Olive oil constituents such as oleuropein (blue) and oleic acid (green) incorporate into the lipid bilayer, altering membrane fluidity and microdomain organization. These modifications influence ion channel gating or distribution (red), thereby affecting intracellular Ca^2+^, Na^+^, and K^2+^ levels. The resulting changes in ion homeostasis modulate cellular signaling, inflammation, metabolism, and excitability, ultimately impacting disease outcomes, including cardiovascular, neurodegenerative, and metabolic disorders.

Olive oil, a hallmark of the Mediterranean diet, is a complex mixture rich in bioactive compounds. Oleic acid and its derivatives, along with some phenolic compounds such as oleocanthal, oleacein oleuropein, hydroxytyrosol, and tyrosol, have emerged as significant modulators of cellular membrane structure and ion channel function [29,42,43,44,45,46]. Although direct data on oleacein’s interaction with specific ion channels are currently lacking, its capacity to strongly integrate into lipid membranes and exert potent antioxidant and anti-inflammatory effects [47], supports a plausible indirect role in influencing redox-sensitive membrane dynamics and ion channel functions. These compounds interact with the lipid bilayer or directly influence ion channel activity [48], thereby affecting physiological processes ranging from neural excitability to cardiovascular function (Figure 2).

### 2.1. Biochemical Mechanisms Underlying the Modulatory Effects of Olive Oil Lipids on Membrane Structure and Ion Homeostasis

Oleic acid plays a fundamental role in the structure and function of cell membranes. Its presence as a free fatty acid (FFA) in biological membranes, although at relatively low concentrations, is particularly relevant in tissues with high transport activity, such as the small intestine. Membrane composition is strongly influenced by dietary fat intake; diets rich in oleic acid (OA) are associated with increased OA levels in various plasma membranes. These changes in lipid composition are accompanied by modulations in the function of several membrane proteins [17].

Structurally, OA promotes the formation of inverted hexagonal phases by interacting with phospholipids such as phosphatidylethanolamine, generating negative curvature that facilitates processes such as membrane fusion and fission. This unique property is attributed to its boomerang-shaped molecular structure, resulting from its cis double bond configuration, which distinguishes it from other fatty acids [49].

Olive oil may also exert favorable effects on lipid peroxidation and oxidative stress, inflammatory responses, and hepatobiliary function compared to other edible oils [50,51].

Oleic acid and its endogenous components, particularly oleoylethanolamide (OEA), have attracted increasing attention due to their diverse physiological roles in metabolism, inflammation, and cellular signaling. Beyond their well-characterized interactions with nuclear receptors such as peroxisome proliferator-activated receptor alpha (PPAR-α), these lipids exert direct biophysical effects on cellular membranes that have profound consequences for ion channel behavior and intracellular ion homeostasis [52,53].

#### 2.1.1. Membrane Integration and Remodeling of the Lipid Microenvironment

OEA, like its precursor oleic acid, is amphipathic and readily incorporated into biological membranes. Upon integration into the phospholipid bilayer, these molecules influence membrane fluidity, curvature stress, lateral pressure profiles, and domain organization. These properties are critical for modulating the function of membrane proteins, particularly voltage-gated ion channels [54,55]. A plausible mechanistic cascade has been proposed to explain how oleic acid derivatives modulate ion channel function and intracellular ion homeostasis. This cascade unfolds through the following sequence of physical and biochemical interactions:

*Membrane Incorporation*: Oleic acid and OEA integrate into the inner leaflet of the plasma membrane either by passive diffusion or via specific transport mechanisms, such FFA- transport proteins, which mediate the uptake and intracellular trafficking of long-chain fatty acids [52,56].

*Bilayer Remodeling*: Upon incorporation, these lipids alter membrane properties by modulating lipid-lipid and lipid-protein interactions. This remodeling affects bilayer thickness, fluidity, and curvature stress, which are critical for the proper localization and function of ion channels [54,55].

*Ion Channel Allosteric Modulation*: Changes in the lipid environment induce allosteric effects on transmembrane proteins, particularly voltage-gated ion channels. Experimental evidence has shown that oleic acid increases the open probability of Kv type channels and stabilizes inactivated states, particularly in neuronal and cardiac models [48,57,58].

*Ion Flux Alteration*: Altered gating kinetics affect the transmembrane movement of ions, such as Na^+^, K^+^, and Ca^2+^. These fluxes influence membrane potential, excitability, and various intracellular signaling cascades [59,60,61].

*Secondary Signaling Activation*: Changes in cytosolic Ca^2+^ levels activate calcium-dependent proteins and pathways, including calmodulin, protein kinase C (PKC) and calcineurin. These secondary messengers influence gene expression, metabolic activity and apoptotic signaling [62,63].

Together, these sequential processes highlight the ability of oleic acid derivatives to modulate both the physical landscape of the membrane and the downstream signaling systems that govern cellular homeostasis.

#### 2.1.2. Intracellular Calcium Dynamics and Organelle Cross-Talk

In addition to their biophysical effects on the plasma membrane, oleic acid derivatives, particularly OEA, modulate intracellular calcium (Ca^2+^) homeostasis. Several studies suggest that these lipids affect key components of Ca^2+^ signaling pathways.

For instance, Wheal et al. [64] showed that OEA—an endogenous ethanolamide synthesized in tissues from oleic acid (the main fatty acid in olive oil) via enzymatic amidation—modulates intracellular Ca^2+^ dynamics in rat vascular smooth muscle. In isolated mesenteric arterial beds and thoracic aortic rings, OEA dose-dependently relaxed vessels and, importantly, suppressed caffeine-induced contractions in Ca^2+^-free buffer. This inhibition of sarcoplasmic/reticulum-mediated Ca^2+^ release indicates that OEA interferes with endoplasmic reticulum Ca^2+^ mobilization, thereby attenuating intracellular Ca^2+^ signaling and contributing to reduced vascular tone [64]. Additionally, Gherardi et al. [65] demonstrated that oleuropein—a major phenolic from olive oil—binds to the MICU1 regulatory subunit of the mitochondrial calcium uniporter complex and directly enhances mitochondrial Ca^2+^ uptake. This activation boosts mitochondrial respiration and ATP production, improving muscle performance and resilience against age-related decline.

Collectively, these studies position oleic acid derivatives as powerful architects of the membrane’s physical terrain and as catalysts of the signaling networks that sustain cellular equilibrium. By softening bilayer rigidity and reshaping lipid microdomains, these lipids not only fine-tune ion channel behavior but also set in motion downstream pathways with far-reaching physiological consequences. For example, by elevating the K^+^-channel open probability and stabilizing inactivation, oleic acid-derived amides can dampen neuronal hyperexcitability, offering a protective buffer against excitotoxic damage in neurodegenerative settings [53]. In cardiac myocytes, enhanced Ca^2+^-handling—mediated through both plasma membrane and mitochondrial mechanisms—appears to stabilize action-potential dynamics and mitigate arrhythmogenic triggers under stress. Simultaneously, by modulating store-operated Ca^2+^ entry and mitochondrial buffering capacity, these compounds bolster insulin responsiveness, elevate energy expenditure and optimize lipid metabolism, underscoring their promise in battling obesity and metabolic syndrome [52]. In this way, the multifaceted actions of oleic acid derivatives bridge membrane biophysics with cellular signaling to safeguard excitability, metabolism, and stress resilience.

### 2.2. Polyphenols: Antioxidant Activity and Ion Channel Interaction

Polyphenols from olive oil including flavonoids, phenolic acids, and secoiridoids such as oleuropein, extend beyond their antioxidant role of neutralizing free radicals. At the membrane interface, oleuropein acts as a primary defender, intercepting ROS before they trigger lipid peroxidation, thereby preserving bilayer integrity and ensuring optimal ion channel function. In models of cardiac ischemia–reperfusion, oleuropein’s dual protection of L-type Ca^2+^-channel gating and mitochondrial membrane potential prevents calcium overload and reduces cell death [66,67].

When oleuropein loses its sugar moiety, forming OLEA, it embeds more deeply into lipid bilayers, particularly those rich in negatively charged lipids, altering local curvature and lipid–protein interactions that are critical for channel clustering, as shown in biophysical and molecular dynamic studies [40,68]. Downstream metabolites like hydroxytyrosol further enhance vascular health by suppressing ROS-driven activation of TRPM2 channels in endothelial cells, mitigating H**_2_**O**_2_**-induced calcium surges, and preserving barrier function and vessel tone [69,70]. Beyond antioxidant effects, oleuropein and OA act as mild agonists of the TRPA1 and TRPV1 receptors, key regulators of energy balance in brown adipose tissue. In obese rats, dietary oleuropein increased noradrenaline, epinephrine and UCP1 levels in BAT, reducing visceral fat and leptin; while OA amplified norepinephrine release, promoting thermogenesis and fat loss via TRP-mediated signaling [71].

Finally, these olive oil phenols leave an imprint on electrically excitable cells. By safeguarding membrane structure and subtly adjusting potassium channel kinetics, they temper neuronal hyperactivity and erect a barrier against excitotoxic insults. Polyphenols found in olive oil, such as oleuropein and oleocanthal, exhibit neuroprotective properties by modulating neuronal excitability and mitigating excitotoxicity. These effects are achieved through antioxidant and anti-inflammatory mechanisms, as well as the modulation of signaling pathways like BDNF/CREB/Akt, which collectively help preserve neuronal integrity [72,73,74]. In the heart, their antioxidant stewardship of the bilayer and direct modulation of both L-type and store-operated calcium channels enhance the steadiness of calcium cycling, reducing the arrhythmogenic sparks that arise under oxidative or ischemic challenge [66]. Simultaneously, through synergy with TRP receptors, SOCE machinery and mitochondrial membranes, these compounds bolster insulin sensitivity, ramp up energy expenditure and fine-tune lipid metabolism—underscoring their compelling therapeutic promise for obesity and metabolic syndrome [52].

### 2.3. Emerging Olive Oil-Derived Compounds and Their Ion-Regulating Potential

Emerging olive oil derivatives, such as ligstroside, oleacein, and oleocanthal, are gaining attention for their membrane-stabilizing, anti-inflammatory, and anti-aggregant properties, which may indirectly influence ion transport mechanisms. Although direct studies on ion channels are limited, oleocanthal, structurally similar to ibuprofen, modulates inflammatory pathways that affects ion channel expression and trafficking [75]. Both oleocanthal and oleacein form adducts with molecules containing primary amine groups, exhibiting antioxidant and anti-inflammatory effects, despite the instability of these adducts limiting their pharmacological applications [76]. Ligstroside, tested at low doses in a cellular model of early Alzheimer’s disease, improved mitochondrial bioenergetics and membrane mechanisms, demonstrating potential in combating mitochondrial dysfunction [77].

Phenolic compounds can be integrated into lipid rafts, influencing the localization and function of signaling molecules and ion channels, such as TRP, BKCa, and KATP channels. These interactions are particularly relevant in vascular physiology and neuroprotection, where subtle changes in ion flux can significantly impact tone, excitability, and survival. Table 2 summarizes research on olive oil compounds modulating ion channel biophysical activity; however, studies on ion transport proteins extend beyond ion channels.

Olive oil and its derivatives exert diverse modulatory effects on ion channels, often in a cell-type- and context-dependent manner. These effects include direct interactions with channel proteins, alterations in membrane composition, and activation of intracellular signaling pathways.

*Cyclic nucleotide-gated (CNG) channels.* In retinal degenerative diseases, oleic acid promotes the functional rescue of CNG channels that are essential for phototransduction under dark conditions and critical for initiating visual signals in photoreceptors. Our laboratory demonstrated that oleic acid enhances membrane trafficking and increases current amplitude in mutant CNG channels associated with inherited retinopathies, such as retinitis pigmentosa and achromatopsia. Using HEK293 cells and whole-cell patch-clamp recordings, we found that oleic acid acts as a chemical chaperone, likely facilitating correct folding, assembly, and surface expression of these channels. These effects align with its known influence on lipid homeostasis and the endoplasmic reticulum stress response, suggesting a therapeutic potential for channelopathies affecting photoreceptor function [2].

*Nicotinic Acetylcholine Receptors (nAChRs*). nAChRs mediate fast synaptic transmission at neuromuscular junctions and in the central nervous system, playing a key role in cognition and motor control. Oleic acid modulates nAChRs through both direct and indirect mechanisms. Antollini and Barrantes [79] used fluorescent probes to demonstrate that oleic acid interacts with transmembrane segments of nAChRs in Torpedo membranes. Molecular dynamics simulations further support this interaction, suggesting that oleic acid can act as a channel blocker [80]. Conversely, in *Xenopus oocytes*, oleic acid enhances acetylcholine-evoked currents via Ca^2+^/calmodulin-dependent protein kinase II (CaMKII), indicating a dual modulatory role [78].

*BK_Ca_ and K_ATP_ Channels: BK_Ca_ and K_ATP_ channels.* These are essential regulators of vascular tone and blood pressure, responding to intracellular calcium and metabolic state, respectively. EVOO phenols activate BK_Ca_ channels through the PLC–IP**_3_**–Ca^2+^ signaling cascade. D’Agostino et al. [99] and Esposito et al. [81] reported this effect in isolated mesenteric arteries, noting vasodilation independent of endothelial function. Additionally, Nkanu et al. [88] demonstrated that chronic EVOO consumption enhances the hypotensive response to BK_Ca_ and K_ATP_ channel openers, indicating systemic vascular benefits.

*Chloride Channels*: Channels transporting chloride such as ClC, TMEM16A, and CFTR, are critical for fluid secretion, neuronal excitability, and epithelial transport. Tewari et al. [83] first described the fatty acid-mediated regulation of ClC-2 activity, which was later confirmed by Cuppoletti et al. [84]. Oleic acid irreversibly blocks TMEM16A (ANO1) in a dose- and voltage-dependent manner at low intracellular Ca^2+^ concentrations [96]. Furthermore, linoleic and oleic acids inhibit CFTR in transfected cells [82], suggesting that dietary lipids may influence secretory processes.

*Human ether-à-go-go-related gene (hERG) Channels*: The hERG potassium channel is essential for cardiac repolarization; its blockade is a common cause of drug-induced arrhythmias. Phenolic compounds from EVOO, such as hydroxytyrosol, do not inhibit hERG currents. Instead, Pitsillou et al. [85] reported that these phenols may enhance the action of antiarrhythmic agents like verapamil, underscoring a favorable cardiac safety profile.

*Voltage-Gated Potassium Channels (Kv):* Kv channels are central to setting membrane potential and shaping action potentials in neurons and cardiac cells. In human atrial myocytes, oleic acid inhibits the transient outward potassium current (Ito) without affecting the inward rectifier current (IK), as shown by Crumb et al. [86]. These findings highlight its selective modulation of action potential morphology. Antollini and Barrantes [48] reviewed how free fatty acids modulate Kv channels by interacting at lipid–protein interfaces, with effects dependent on chain length, saturation, and geometry. In hypothalamic pro-opiomelanocortin (POMC) neurons, oleic acid suppresses KATP currents independently of G-protein signaling, an effect reversed by diazoxide, supporting a direct mechanism [100]. In vascular smooth muscle cells, oleic acid downregulates Kir6.1 expression, reducing ATP-sensitive K^+^ currents and potentially altering vascular tone [3]. Chronic EVOO intake also sensitizes K_ATP_ and BK_Ca_ channels, as shown in vivo by Nkanu et al. [88], who observed reduced arterial pressure and an increased responsiveness to channel openers.

*Voltage-gated potassium channel (KCNQ1) Channels*: KCNQ1 channels contribute to the slow delayed rectifier potassium current (IKs), which is essential for cardiac repolarization and arrhythmia prevention. Molecular simulations and experimental data indicate that oleic and linoleic acids interact with the voltage sensor domain via their negatively charged head groups, potentially modulating gating [89]. However, olive oil components exhibit channel-specific effects across the Kv family [90,91,92].

*L-Type Calcium Channels*: L-type Ca^2+^ channels mediate calcium influx in excitable tissues, driving muscle contraction and hormone secretion. Oleuropein, a major olive phenol, decreases the L-type Ca^2+^ current (ICa,L) in neonatal rat cardiomyocytes without proarrhythmic effects in rabbit hearts. These reversible changes suggest potential use in antihypertensive therapies [93].

*Transient Receptor Potential (TRP) Channels*: TRP channels serve as polymodal sensors of temperature, pain, stretch, and chemical stimuli. EVOO components like oleocanthal selectively activate TRPA1, explaining the pungent throat sensation after EVOO ingestion [94]. OLEA activates TRPA1 and TRPV1 in sympathetic neurons and adipose tissue, increasing norepinephrine release and thermogenesis in obese rats [71]. Oleic acid inhibits TRPV1 by stabilizing the closed state and reducing capsaicin-induced activity in dorsal root ganglion neurons and HEK293 cells [95,101]. In immune cells, oleic acid activates TRPC3 and TRPC6, potentially influencing T-cell signaling [51].

*PIEZO Channels*: PIEZO channels are key mechanotransducers in proprioception, baroreception, and touch. The mechanosensitive PIEZO2 channel, involved in tactile perception and motor coordination, is upregulated by a linoleic acid–rich diet. Romero et al. [97] demonstrated that this diet improved mechano-excitability and gait in mice with Angelman Syndrome, suggesting lipid-dependent modulation of PIEZO2.

Voltage-gated sodium channels (Nav): These channels initiate and propagate action potentials in excitable cells. Oleic acid inhibits skeletal muscle sodium channels (hSkM1) expressed in HEK293T cells [98], potentially altering muscle excitability and function. These isoform-specific effects highlight the nuanced interaction between fatty acids and sodium channel gating.

An allosteric modulation of Kv channels through fatty acid binding has also been suggested by structural and biophysical studies, revealing that long-chain fatty acids can bind directly within the central cavity of voltage-gated K^+^ channels, stabilizing nonconductive states and altering gating kinetics. Smithers et al. [102] used the fluorescent probe 11-dansylaminoundecanoic acid (Dauda) to map a high-affinity binding site in the central cavity of the bacterial K^+^ channel KcsA, finding a dissociation constant of 0.47 ± 0.10 µM for Dauda and demonstrating displacement by oleic acid (Kd ≈ 2.9 µM) and other fatty acids via lipid–bilayer access to the closed pore. Displacement by tetrabutylammonium confirmed localization in the cavity, while variations in chain length and unsaturation modulated affinity in line with electrophysiological block observed in mammalian Kv channels. These findings support a model whereby oleic acid and related lipid derivatives partition into the membrane, enter the pore’s hydrophobic cavity, and allosterically stabilize inactivated channel conformations, thereby reducing open probability and prolonging inactivation in neuronal and cardiac Kv channels [102].

### 2.4. Triterpenes in Olive Oil and Their Interaction with Ion Channels

Triterpenes such as oleanolic and maslinic acid are natural pentacyclic compounds found in the non-saponifiable fraction of virgin olive oil, typically in concentrations ranging from 8.9 to 112 mg/kg, depending on the olive variety, degree of ripeness, and processing method [103,104].

Although they are present in low amounts compared to fatty acids and phenolics, these compounds have gained interest for their potential biological activity, including the modulation of ion channels. For instance, oleanolic acid isolated from olive oil has been shown to reduce the activity of the TRPV1 (transient receptor potential vanilloid 1) channel. At a concentration of 90 µM, it significantly inhibited capsaicin-induced currents in electrophysiological recordings, suggesting a stabilizing effect on the closed state of the channel [105].

The increasing popularity of olive oil has sparked interest in how its key components such as oleic acid and other minor compounds modulate cell membranes and ion flow. These compounds alter membrane lipid composition and influence the function of membrane proteins, including ion channels and receptors, affecting essential processes such as ion channel activity, receptor signaling, and protein trafficking.

Research suggests that modifying membrane lipid content can regulate cellular signaling pathways, offering therapeutic strategies targeting plasma and organelle membranes where critical physiological functions occur. Although some therapies are under investigation, the full potential of lipids as lipochaperones and triterpenes or phenolic compounds as cellular modulators remains underexplored.

## 3. Beyond Classical Channels: Modulation of Ion Transport Systems by Olive Oil-Derived Compounds

While ion channels are the primary targets of olive oil-derived compounds, a growing body of evidence indicates that these bioactive lipids also influence a broader range of ion transport systems, including ion pumps, aquaporins, store-operated calcium entry (SOCE) mechanisms, and redox-sensitive membrane proteins. These systems are essential for maintaining osmotic balance, ionic gradients, and proper cellular signaling.

*Na^+^/K^+^-ATPase Inhibition in Pulmonary Tissue*: Oleic acid inhibits the Na^+^/K^+^-ATPase pump in alveolar epithelial cells when administered intratracheally in mice, disrupting fluid reabsorption in the lungs and contributing to pulmonary edema. This inhibition is accompanied by leukocyte activation, lipid body formation, and the release of proinflammatory lipid mediators [106].

*Suppression of Store-Operated Ca^2+^ Entry (SOCE*): In human colorectal adenocarcinoma (HT29) cells, oleic acid significantly reduces SOCE—a crucial mechanism for calcium replenishment in the endoplasmic reticulum. This inhibition may result from direct interference with the STIM1/Orai1 complex or alterations in membrane lipid composition that affect channel assembly and function [107,108].

*Aquaporin Regulation and Water-Glycerol Homeostasis*: Oleic acid modulates water and solute permeability by altering aquaporin expression in hepatocytes. Specifically, it downregulates AQP3 and upregulates AQP9 through activation of the p38 MAPK pathway, affecting hepatic glycerol transport and water balance with implications for lipid accumulation and steatosis development [109].

*Preservation of Redox-Sensitive Transporters*: Regular intake of EVOO enhances antioxidant defenses, as evidenced by elevated levels of superoxide dismutase (SOD) and catalase, and reduced lipid peroxidation in cardiac and renal tissues [88]. This redox balance is critical for preserving the function of redox-sensitive transport proteins, such as ion pumps, exchangers, and voltage-independent Ca^2+^ transporters, which are vulnerable to oxidative inactivation.

*Potential Gap Junction Modulation*: Gap junctions, formed by connexin proteins (e.g., Cx43), facilitate intercellular ion exchange and synchronized electrical activity, particularly in the heart and vasculature. These structures are highly susceptible to oxidative stress. Olive oil phenolics, including hydroxytyrosol and oleuropein, protect connexin integrity under oxidative conditions [110]. Although Nkanu et al. [88] did not directly assess gap junctions, the improved antioxidant status observed in EVOO-fed animals suggest the gap junction communication may be preserved, potentially enhancing coordinated vasodilatory responses [88].

Additionally, Fujiwara et al. [108] demonstrated that oleuropein improves insulin resistance in skeletal muscle by promoting the translocation of the glucose transporter GLUT4 to the cell membrane. This effect enhances glucose uptake in muscle cells, which is a key mechanism for improving insulin sensitivity. The study suggests that oleuropein could have potential benefits for managing metabolic disorders such as type 2 diabetes [111].

## 4. Conclusions and Perspectives

Experimental findings underscore the diverse mechanisms by which olive oil constituents modulate ion transport. Oleic acid and related lipids act on various ion channels, either enhancing or inhibiting their function depending on cellular context. Meanwhile, olive oil phenolics often preserve or fine-tune ion channel activity, particularly under stress conditions. These effects contribute to the sensory, metabolic, and protective roles in cardiovascular, neural, and immune health. As research progresses, olive oil emerges not only as a dietary staple but also as a molecular modulator of bioelectric signaling.

The evidence supports a unified conceptual framework in which olive oil-derived lipids act as multimodal regulators of bioelectric signaling. Rather than targeting a single molecular entity, these compounds influence ion transport through mechanisms intersecting membrane physiology and cellular excitability.

Central to this framework is the ability of oleic acid and its derivatives to alter membrane biophysics, modifying lipid packing, curvature, and microdomain organization. These structural changes affect the function of embedded proteins, including voltage- and ligand-gated ion channels, aquaporins, and redox-sensitive transporters. Additionally, olive oil phenolics and fatty acid amides modulate the activity and expression of non-channel transport systems, such as the Na^+^/K^+^-ATPase, store-operated calcium entry (SOCE) mechanisms, and glucose transporters like GLUT4.

This multifaceted regulatory network affects cellular ion flux, electrical potential, osmotic homeostasis, and intracellular signaling. These effects are context-dependent, varying with tissue type, oxidative status, and metabolic demands. For example, oleuropein’s enhancement of GLUT4 translocation in skeletal muscle improves insulin sensitivity, whereas Na^+^/K^+^-ATPase inhibition in alveolar cells may exacerbate inflammation under pathological conditions.

This integrative view, as approached in this review, allows us to appreciate olive oil not only as a staple of traditional diets, but as a biological toolkit with the potential to support cardiovascular, neurological, and metabolic health. It invites us to see nutrition as a form of molecular dialog between our food and our physiology. This perspective not only deepens our understanding of olive oil’s biological impact but also lays the groundwork for future therapeutic strategies targeting disrupted ion homeostasis and electrical signaling in major chronic diseases.

## Figures and Tables

**Table 1:** Main chemical constituents of olive oil.

Fraction	Category	Representative Compounds
Saponifiable (98–99%)	Fatty acids (as triacylglycerols)	Oleic acid (C18:1), Palmitic acid (C16:0), Linoleic acid (C18:2), Stearic acid (C18:0), Palmitoleic acid (C16:1)
Non-saponifiable (1–2%)	Sterols (lipidic)	β-Sitosterol, Campesterol, Stigmasterol
	Hydrocarbons (lipidic)	Squalene
	Triterpenes	Oleanolic acid, Maslinic acid, Uvaol
	Tocopherols (Vitamin E)	α-Tocopherol
	Simple phenols	Hydroxytyrosol, Tyrosol
	Secoiridoids	Oleuropein aglycone, Ligstroside aglycone, Oleocanthal, Oleacein, Oleomissional, Oleocoronal, Oleocanthalic acid
	Phenolic acids	Caffeic acid, Vanillic acid, Ferulic acid
	Flavonoids	Luteolin, Apigenin
	Lignans	Pinoresinol, Acetoxypinoresinol
	Pigments	Chlorophylls, Carotenoids
	Volatile compounds	Aldehydes, ketones, esters

Note: In this table, the term “non-saponifiable fraction” follows the operational definition used in standard analytical procedures for olive oil, referring to the fraction recovered after saponification. It includes bioactive minor components such as phenolic compounds (e.g., hydroxytyrosol, oleuropein aglycone, caffeic acid, luteolin, pinoresinol), tocopherols (vitamin E), triterpenes in their free acid or alcohol forms (e.g., oleanolic acid, maslinic acid, uvaol), squalene, carotenoids, and sterols. Although some of these molecules—like triterpenic acids—are chemically capable of saponification if esterified, they are present in olive oil in free form and thus appear in the non-saponifiable fraction. These compounds are associated with antioxidant, anti-inflammatory, cardioprotective, and other health-promoting effects.

**Table 2:** Studies on phenolic and lipidic olive oil compounds modulating ion channel biophysical activity.

Class	Compound(s)	Ion Channel	Biophysical/Physiological Effect	Reference
Lipidic	Linoleic Linolenic acid Oleic acid	ACHR Acetilcholine receptor	-Linoleic and linolenic acid enhances ACh-gated currents via CaMKII activation in *Xenopus* oocytes. -Using the fluorescent probe suggests that there is a direct lipid-protein influence. -Acts as a channel blocker, inhibiting nicotinic acetylcholine receptor function.	[78,79,80]
Phenolic	EVOO	BKCa channels Large-conductance Ca^2+^-activated K^+^ channel	EVOO phenols activate BKCa channels in smooth muscle of uterine arteries of pregnant rats, mediated by Ca^2+^ signaling that triggers the synthesis of NO, cGMP and opening of BKCa.	[81]
Lipidic	Oleic and Linoleic acid	CFTR Cystic fibrosis transmembrane receptor	Using patch clamp recording from CFTR-transfected baby hamster kidney cell lines, the CFTR was inhibited by several fatty acids in the following order: linoleic ≥ arachidonic ≥ oleic.	[82]
Lipidic	Oleic acid	ClC2	Patch clamp on HEK-293 cells demonstrated activation of ClC-2 by oleic acid.	[83,84]
Lipidic	Oleic acid	CNG Cyclic Nucleotide-Gated	Facilitates trafficking and function of mutant CNG channels related to retinopathies.	[2]
Phenolic	Oleuropein, Hydroxytyrosol	ERG Ether-à-go-go-Related Gene	Considered non-inhibitors of hERG channels; may enhance the action of verapamil, suggesting a favorable cardiac safety profile.	[85]
Lipidic	Oleic acid	IK Inward rectifying potassium channel	It blocks the transient outward current (Ito) in human atrial myocytes, while leaving the sustained current and inward rectifier current (IK1) unchanged.	[86]
Lipidic	Oleic acid	KATP ATP-sensitive potassium	-Inhibits protein expression and current in human umbilical artery smooth muscle cells, potentially affecting vascular tone. -Inhibits KATP currents in pro-opiomelanocortin (POMC) neurons, influencing neuronal excitability.	[87]
Phenolic	EVOO phenols	K_ATP_ and BK_Ca_ ATP-sensitive potassium Large-conductance Ca^2+^-activated K^+^ channel	Activation of K_ATP_ and BK_Ca_ channels in vascular smooth muscle, causing cellular hyperpolarization, vasorelaxation and reduction in mean arterial pressure.	[88]
Lipidic	Linoleic acid	KCNQ1	Provide molecular models supported by experimental evidence of specific interactions between PUFA analogs and KCNQ1 channel.	[89]
Lipidic	Oleic acid/Linoleic acid	Kv1.3	Whole-cell patch-clamp experiments demonstrated that the polyunsaturated linoleic acid decreased the activation and inactivation time constants of the Kv1.3 channels, but did not affect the voltage dependence of the steady-state activation and steady-state inactivation of the channels, while monounsaturated oleic acid did not result in significant changes in the biophysical parameters.	[90]
Lipidic	Oleic acid	Kv7.1	Oleic acid has no effect on channel kinetic.	[91]
Lipidic	Oleic acid	Kv7.2/3	Oleic acid did not facilitate opening of the human Kv 7.2/3 channel expressed in *Xenopus oocytes.*	[92]
Phenolic	Hydroxytyrosol	L-type Ca^2+^ channels	Direct and reversible blockade of L-type Ca^2+^ channels in vascular smooth muscle, in a dose-dependent manner, reducing vascular resistance and contributing to vasodilation.	[93]
Phenolic	Oleocanthal	TRPA1	Selectively activates TRPA1 channels, contributing to the pungent oral sensation of extra virgin olive oil.	[94]
Phenolic	Oleuropein aglycone	TRPA1/TRPV1	OA is the agonist of both TRPA1 and TRPV1	[71]
Lipidic	Oleic acid	TRPV1	Reduces open probability by stabilizing the closed state; slight antagonism to capsaicin activation.	[95]
Lipidic	Oleic acid	TRPC3/6	Increases intracellular Ca^2+^ in T-cells via activation of TRPC3/6 channels, influencing immune cell function.	[50]
Lipidic	Oleic acid	TMEM16A Transmembrane member 16A	Irreversibly blocks the channel in a dose- and voltage-dependent manner at low intracellular Ca^2+^ concentrations.	[96]
Lipidic	A linoleic acid	PIEZO2 Stretch-gated ion channel	LA-enriched diet increases PIEZO2 activity, mechano-excitability, and improves gait in male mice with Angelman Syndrome. Whole-cell recordings post-mechanical stimulation confirmed increased responses and improved gait.	[97]
Lipidic	Oleic acid	SkM1 skeletal muscle sodium channels	Currents from SkM1 transfected into HEK293t cells were inhibited by oleic acid	[98]

## Data Availability

No new data were created or analyzed in this study. Data sharing is not applicable to this article.

## Abbreviations

The following abbreviations are used in this manuscript:

Abbreviation	Concept
ACHR	Acetylcholine Receptor
AQP	Aquaporin
ATP	Adenosine Triphosphate
BAT	Brown Adipose Tissue
BKCa	Calcium Activated Potassium Channel
CaMKII	Ca^2+^/Calmodulin-Dependent Protein Kinase II
CFTR	Cystic Fibrosis Transmembrane Conductance Regulator
cGMP	Cyclic Guanosine Monophosphate
CNG	Cyclic Nucleotide-Gated
DAG	Diacylglycerols
ER	Endoplasmic Reticulum
EVOO	Extra Virgin Olive Oil
FFA	Free Fatty Acid
GLUT	Glucose Transporter
HEK	Human Embryonic Kidney Cells
hERG	Human Ether-A-Go-Go Related Gene
hSkM1	Skeletal Muscle Sodium Channels
IR	Inward Rectifier Current
IOC	International Olive Council
KATP	Atp-Sensitive Potassium Channel
Kv	Voltage-Gated Potassium
LDL	Low-Density Lipoproteins
MAG	Monoacylglycerols
MAPK	Mitogen-Activated Protein Kinase
MUFA	Monounsaturated fatty acid
nAChRs	Nicotinic Acetylcholine Receptors
Nav	Voltage-Gated Sodium Channels
OEA	Oleoylethanolamide
OLEA	Oleuropein Aglycone
PIEZO	Mechanosensitive Ion Channel Protein
PKC	Protein Kinase C
POPC	1-palmitoyl-2-oleoyl-sn-glycero-3-phosphocholine
POPG	1-Palmitoyl-2-oleoyl-sn-glycero-3-phosphoglycerol
POMC	Pro-Opiomelanocortin
PPAR-α	Peroxisome Proliferator-Activated Receptor Alpha
PUFA	Polyunsaturated fatty acid
ROS	Reactive Oxygen Species
SGLT1	Sodium-Glucose Linked Transporter
SOCE	Store-Operated Calcium Entry
SOD	Superoxide Dismutase
STIM1	Stromal Interaction Molecule 1
TAG	Triacylglycerols
TRPA1	Transient Receptor Potential Ankyrin 1
TRPM2	Transient Receptor Potential Melastatin 2
TRPV1	Transient Receptor Potential Vanilloid 1
UCP1	Uncoupling Protein 1
